# SARS-CoV-2 Infection during Pregnancy and Histological Alterations in the Placenta

**DOI:** 10.3390/diagnostics12092258

**Published:** 2022-09-19

**Authors:** Irina Pacu, George-Alexandru Roșu, Giorgia Zampieri, Anca Rîcu, Alexandra Matei, Ana-Maria Davițoiu, Teodora Vlădescu, Crîngu Antoniu Ionescu

**Affiliations:** 1 Faculty of Medicine, “Carol Davila” University of Medicine and Pharmacy, 050474 Bucharest, Romania; 2 Department of Obstetrics and Gynecology, “Sfântul Pantelimon” Clinical Emergency Hospital, 021659 Bucharest, Romania; 3 “Dr. Victor Gomoiu” Children’s Clinical Hospital, 022102 Bucharest, Romania; 4 Pathological Anatomy Department, “Sfântul Pantelimon” Clinical Emergency Hospital, 021659 Bucharest, Romania

**Keywords:** SARS-CoV-2, placenta, pregnancy, newborn

## Abstract

(1) Background: Despite the high number of cases of COVID-19 during pregnancy, SARS-CoV-2 congenital infection is rare. The role of the placenta as a barrier preventing the transmission of SARS-CoV-2 from the mother to the fetus is still being studied. This study aimed to evaluate the impact of SARS-CoV-2 infection on placental tissue. (2) Methods: This was a transversal monocentric observational study. In the study, we included pregnant women with COVID-19 who delivered at “Sfântul Pantelimon” Clinical Emergency Hospital between 1 April 2020 and 30 March 2022. Histological analyses, both macroscopic and microscopic, were performed for placentas that came from these cases. (3) Results: To date, a characteristic placental lesion has not been clearly demonstrated, but most findings include features of maternal and fetal vascular malperfusion, which probably reflect the reduction in placental blood flow due to low oxygen level from the hypoxic respiratory disease and underlying hypercoagulable state induced by the COVID-19 infection. (4) Conclusions: The histopathological aspects found in placentas that came from COVID-19-positive pregnant women are common for many other diseases, but when they are found together, they are highly suggestive for viral infectious involvement of the placenta.

## 1. Introduction

The SARS-CoV-2 (severe acute respiratory syndrome coronavirus 2) is an enveloped single positive-stranded ribonucleic acid (RNA) virus belonging to the coronavirus family, responsible for the current pandemic of acute respiratory infections worldwide, also known as COVID-19 (Coronavirus disease 2019) [1]. The first cases of the disease were found in Wuhan, China, in 2019 [1]. After spreading rapidly to other countries, COVID-19 was declared a Public Health Emergency of International Concern [2,3,4].

The known transmission pathways include the inhalation of tiny droplets, close contact with virus carriers, contact with a surface contaminated by SARS-CoV-2 and aerosol transmission [5]. The symptoms include fever, cough, shortness of breath, muscle pain, fatigue and gastrointestinal disturbances [6]. In severe cases, it can cause pneumonia, acute respiratory distress syndrome, sepsis, septic shock and a cytokine storm with multiorgan failure and disseminated coagulopathy, leading to death [6]. Furthermore, SARS-CoV-2 infection can cause non-respiratory complications—brain haemorrhage, memory loss, altered mental states in both sexes, semen quality worsening in male patients [7,8] and pregnancy complications such as preeclampsia [9].

The impact of COVID-19 on pregnant women is a key area of interest.

Several studies concluded that the severity of COVID-19 in pregnant women depends on comorbidities such as age over 34, body mass index (BMI) above 35, gestational diabetes and hypertension [10].

Congenital infection of SARS-CoV-2 appears rare, despite many cases of COVID-19 during pregnancy [11,12,13,14]. Even though the syncytiotrophoblast, which lines the surface of the placenta, provides a good barrier to placental infection, vertical transmission to the foetus can happen [11,14]. In these cases, the virus enters the foetal bloodstream by direct placental infection or by foetal swallowing or aspiration of infected amniotic fluid [11,12,13,14].

Cytomegalovirus (CMV) and herpes simplex virus (HSV) infections, both deoxyribonucleic acid (DNA) viruses, lead to histological signs of chronic inflammation of/in the villi, known as chronic villitis, or the intervillous space, known as intervillositis [11,15]. In some cases, both types of histological aspects of chronic inflammation can be seen [11,16]. In patients infected with Zika and Dengue virus, both RNA viruses, intervillositis was observed [16]. It was also concluded that in Zika virus infection, proliferation of Hofbauer cells (specialized placental macrophages) can be found [17].

SARS-CoV-2 infects the tissues via angiotensin-converting enzyme 2 (ACE2), its specific receptor, while entry into the cell requires spike protein cleavage by the transmembrane serine protease 2 (TMPRSS2) [18].

Histopathological examination of the placenta from COVID-19-positive mothers can provide significant information regarding the coronavirus’s effect on maternal and foetal outcomes. Several studies have already analysed the histology of the placentas from COVID-19 mothers, describing primarily microvascular changes, while an inflammatory response was occasionally encountered [19,20,21,22].

The main aim of this work was to evaluate the impact of SARS-CoV-2 infection on placental tissue.

## 2. Materials and Methods

We conducted a transversal, observational, monocentric and retrospective study, analysing the cases of pregnant women admitted to the Department of Obstetrics and Gynecology from “Sfântul Pantelimon” Clinical Emergency Hospital from Bucharest, Romania, during a two-year timeframe, from 1 April 2020 to 30 March 2022, that were positive for SARS-CoV-2 infection at the moment of admission and gave birth during their hospitalisation, in the first 14 days after the COVID-19 diagnosis. The objectives of this study were to identify if there are specific histopathological anomalies related to SARS-CoV-2, the degree of placental impairment and the severity of this injury in relation with the severity of maternal COVID-19.

Maternal SARS-CoV-2 infection was diagnosed on the basis of the results of the nasopharyngeal sample collected at the time of hospital admission and a positive result validated by an accredited laboratory. Patients who were diagnosed positive for SARS-CoV-2 at the time of birth and were also positive in the first trimester and/or the second trimester of pregnancy were excluded from the study to isolate the effects of infection per trimester. All the newborns from COVID-19-positive mothers were tested for SARS-CoV-2 infection through reverse transcription polymerase chain reaction (RT-PCR) (nasopharyngeal sample) on the first day after birth. The placentas were not tested for SARS-CoV-2 infection.

In all cases, we analysed the maternal and newborns’ characteristics, COVID-19 severity, placental injury due to SARS-CoV-2 infection and the correlation between those aspects. The systemic inflammatory and hypercoagulable state was analysed for every patient. Maternal and newborns’ characteristics were obtained from the patients’ observation sheet. Macroscopic and microscopic descriptions of each placenta were gathered from the histopathological reports provided by the Anatomical Pathology Department of our Hospital.

SARS-CoV-2 infection at the time of birth was classified in the following categories [23,24]:

- Asymptomatic if the PCR test was positive but without respiratory or general symptoms;
- Mild if there were any of the following signs or symptoms: fever, chills, mild cough, headache, etc., but without shortness of breath, chest pain, or breathlessness;
- Moderate if there were respiratory difficulties, suggestive pulmonary imaging, and/or peripheral capillary oxygen saturation (SpO2) > 94%;
- Severe if the respiratory rate was greater than 30 breaths per minute, SpO2 < 94%, severe breathlessness, cough, altered general condition, and severe respiratory failure.

For all placentas, a histopathological analysis was performed both macroscopically and microscopically to identify the pathological findings. The microscopic pathological aspects that were searched were selected from the ones that were found most frequently in other studies that had the same objectives as our study from the literature [2,6,13,25,26,27,28,29,30]. The slides were examined on Haematoxylin and Eosin staining and photographed on a Leica DM750 Microscope. The placentas were not tested for SARS-CoV-2 through RT-PCR.

The patients gave their informed consent for the use of their and their newborn’s data on studies at the moment of the admission to the hospital. The study received the ethical approval from the Ethical Commission of the “Sfântul Pantelimon” Clinical Emergency Hospital Bucharest (approval code 12878/28.06.2022) and the study was conducted according to the guidelines of the Declaration of Helsinki.

## 3. Results

This monocentric study has analysed, retrospectively, all the admissions to the Department of Obstetrics and Gynecology from the “Sfântul Pantelimon” Clinical Emergency Hospital Bucharest from 1st of April 2020 until 30th of March 2022, and searched for all cases that had as main discharge diagnosis “vaginal/cesarean section delivery” and as secondary diagnosis “COVID-19”. During the 2-year timeframe, 50 pregnant women were admitted in the Clinic for various symptoms (bleeding, amniotic fluid leakage, uterine contractions) and they gave birth during their hospitalisation, being diagnosed or reconfirmed peripartum with SARS-CoV-2 infection.

**Figure 1:**
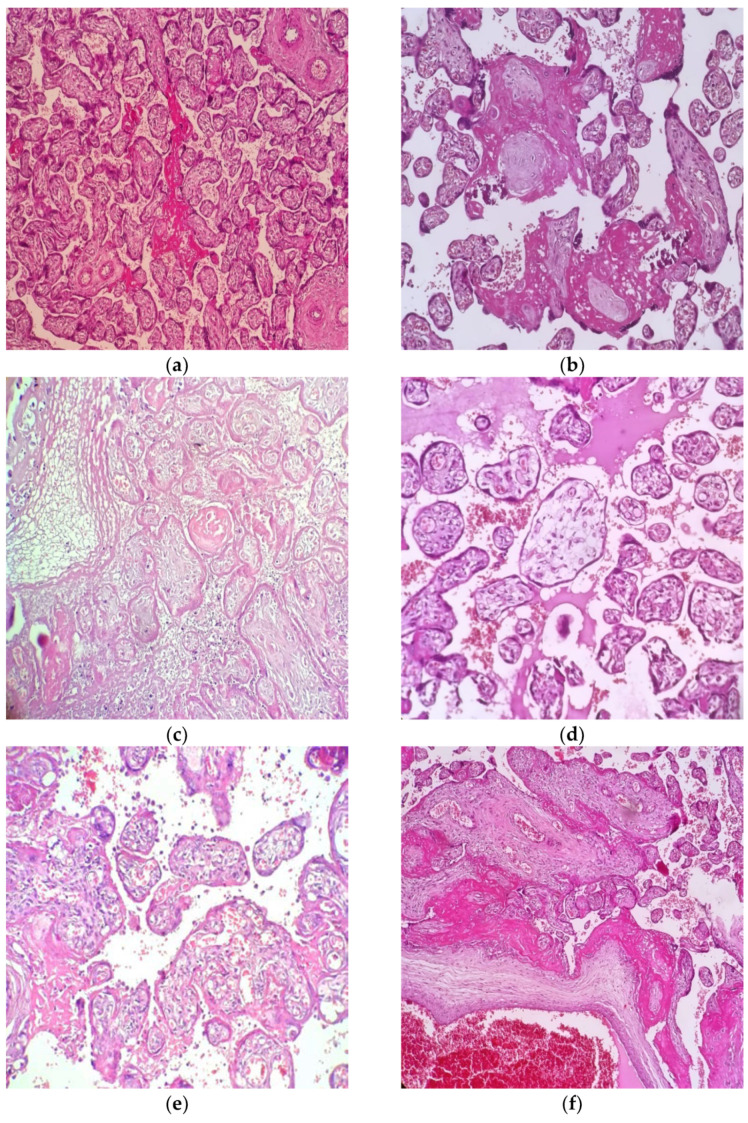
Microscopic aspects most frequently identified in placentas from SARS-CoV-2-positive pregnant women—Haematoxylin and Eosin staining, Leica DM750 Microscope. (**a**) Perivillous fibrin—10× magnification; (**b**) Avascular villi—20× magnification; (**c**) Villous infarction and necrosis—20× magnification; (**d**) villous oedema—20× magnification; (**e**) Villitis—20× magnification; (**f**) Increased perivillous fibrin deposits and intervillous thrombosis—10× magnification.

For these patients, the elements related to their demographics, pregnancy and labour characteristics were analysed (Table 1). After birth, the newborns were characterised by their birth weight, correlation with the estimated foetal weight for gestational age, Apgar score, SARS-CoV-2 infection status and clinical course until discharge (Table 2). The third element that was investigated is represented by the placentas’ characteristics, both macroscopic and microscopic, for signs of maternal and foetal vascular malperfusion and other elements that could suggest this structure’s involvement in relation with the SARS-CoV-2 infection (Table 3, Figure 1).

### 3.1. Maternal Characteristics

During the 2-year timeframe, the hospital protocol stated that every pregnant woman who gave birth in the Obstetrics and Gynecology Department, who proved to be positive for SARS-CoV-2 infection, should be transferred to the designated hospitals that dealt exclusively with COVID-19 patients. For this reason, many patients who gave birth in our unit were transferred to Bucur Maternity, with their newborns, in the first 24 h (hour) after birth, resulting in insufficient data regarding postpartum and postnatal course of those patients and their newborns.

Analysing the number of follow-up examinations for the patients included in the study, we discovered that the average number is 4.4, with values situated between 0 and 12. A total of 15 patients (30% of cases) did not have any obstetrical examinations during their pregnancy (Table 1).

The average gestational age at the moment of the COVID-19 diagnosis in this study was 38 weeks of gestation, extending from 27 weeks of gestation up to 41 weeks of gestation.

The interval between the RT-PCR SARS-CoV-2-positive result (COVID-19 diagnosis) and birth was on average 0.94 days, varying between 0 days and 12 days, and in the large majority of cases (84% of cases), the delivery occurred on the same day as the COVID-19 confirmation (Table 1).

In this study, we identified 42 patients (84% of cases) with asymptomatic COVID-19, 7 patients with mild disease (14% of cases) and 1 patient with a severe form of COVID-19.

Patients with mild disease presented with dry cough, rhinorrhea and myalgia. The only patient with severe disease was confirmed with SARS-CoV-2 infection 10 days before admission and had a slow degradation of her health until day 10, when she presented with acute pulmonary insufficiency with SpO2 84% with nasal cannula (put on positive pressure ventilation). She delivered through caesarean section 2 days after admission. The main reason for delivery in this case was acute foetal distress with bradycardia. In the postpartum period, the computer tomography scan showed 50% lung damage. Her health worsened during the following days. The patient presented multiple organ failure, was intubated with mechanical ventilation and died 6 days after delivery. The patient with severe symptoms was the only one to use oxygen.

A total of 9 patients (18% of cases) presented pregnancy-related complications such as gestational hypertension (3 cases), foetal growth restriction (1 case), gestational diabetes (1 case), Rh incompatibility with isoimmunisation (2 cases) and Klebsiella vaginosis (2 cases).

C-reactive protein level presented an average value of 53.79 mg/L and a median value of 29.40 mg/L, with values situated between 2.34 mg/L and 198.30 mg/L. These values might not reflect reality because for 28 patients (56% of cases) this analysis was not taken due to hospital protocol regulations at that moment regarding asymptomatic patients, especially at the onset of the pandemic, and because many patients were transferred to the designated hospitals that dealt exclusively with COVID-19 patients at the moment of a SARS-CoV-2-positive result.

In our analysis, we observed that the percentage of vaginal births was 60% (30 cases) and the number of cases that delivered through caesarean section was 20 (40% of cases), in contrast to the proportion of caesarean deliveries in our Clinic, which was around 65–69% each year, for the last 5 years.

The average levels of haematological indicators in this study were found in the normal range—average level of Haemoglobin was 11.85 g/dL (with values between 8.4 g/dL and 14.5 g/dL), average level of leukocytes was 10.90 × 10^9^/L (with values between 6.23 × 10^9^/L and 15.66 × 10^9^/L), average level of Thrombocytes was 255.54 (with values between 114 × 10^9^/L and 426 × 10^9^/L)—without any cases of severe anaemia, leukocytosis, leukopenia, thrombocytosis, or thrombocytopenia when the normal reference ranges of the laboratory values were adjusted for pregnancy.

### 3.2. Newborns’ Caracteristics

All the newborns that were born from SARS-CoV-2-positive women included in this study were alive at the moment of birth. Additionally, all the newborns were tested for SARS-CoV-2 infection and all the results were negative, so no transplacental transmission was identified in our study.

In our study, the average neonatal weight was 3046.8 g, with values situated between 690 g and 3800 g. A total of 48 newborns out of 50 were adequate for gestational age (AGA) (average percentile for neonatal weight 48, median percentile for our study 49, with a minimum of 33 and a maximum of 57, values reported to Caucasian nomograms) and 2 neonates were small for gestational age (SGA) (Table 2).

We had only one neonatal death due to severe neonatal respiratory distress syndrome because of extreme prematurity. This case came from a 27-week pregnant woman who presented with gestational hypertension and severe foetal growth restriction with reversed end-diastolic flow on the umbilical artery Doppler ultrasound.

The amniotic fluid was clear in 42 cases (84% of cases) and meconium-stained in 8 cases. All the newborns with meconium-stained amniotic fluid came from mothers with inadequate prenatal care.

The Apgar score at 1 min (minute) was on average 9, with values varying between 3 and 10. Only one newborn had an Apgar score at 1 min of 3, originating from a pregnant woman who presented without any follow-up visits during pregnancy and had meconium-stained amniotic fluid at birth and systemic infection with Klebsiella.

In the distinct analysis of the number of days the newborns stayed in the neonatal department, an average value of 6.86 days was identified (values varying between 3 days and 17 days) (Table 2). A total of 23 newborns had an uncomplicated clinical course until discharge and 12 newborns (24% of cases) were transferred together with their mothers to the hospitals that dealt exclusively with COVID-19 patients, and they were taken out of the analysis, being lost to follow up. A total of 12 newborns (24% of cases) were admitted to the Neonatal Intensive Care Unit (NICU) due to various pathologies, most frequently for neonatal respiratory distress syndrome (8 newborns, 16% of cases). One newborn presented with aspiration pneumonia due to meconial amniotic fluid and 3 newborns (6% of cases) presented with bacterial sepsis.

### 3.3. Placental Caracteristics

The average weight of the placentas was 509 g, with values situated between 115 g and 633 g (Table 3). A total of 41 placentas (82% of the total number of cases) had a normal macroscopic appearance, 8 were meconium-stained and 1 presented with calcar deposits.

The microscopic pathological aspects were divided into four subcategories: maternal side signs of vascular malperfusion, foetal side signs of vascular malperfusion, inflammatory changes and other placental findings.

The maternal signs of vascular malperfusion were identified in our study with the following frequencies: villous infarction (both central and peripheral) and necrosis (30% of cases), increased perivillous fibrin deposition (64% of cases), accelerated villous maturation (36% of cases), Tenney-Parker change (38% of cases), decidual arteriopathy (seen as atherosis and fibrinoid necrosis, mural hypertrophy of membrane arterioles or absence of spiral artery remodeling) (20% of cases), intervillous thrombosis (46% of cases) and increased microcalcifications (56% of cases) (Figure 1).

The foetal signs of vascular malperfusion most frequently identified in our analysis were represented by the following: avascular villi (72% of cases), delayed villous maturation (34% of cases), thrombi in the foetal circulation (32% of cases), karyorrhexis (22% of cases) and chorangiosis (20% of cases) (Table 3, Figure 1).

The inflammatory changes, although not frequently identified in the placentas that came from SARS-CoV-2-positive pregnant women, were represented by the following: villitis (32% of cases), villous oedema (in 24% of cases), subchorionitis (18% of cases), chorioamniotitis (14% of cases) and chronic deciduitis (14% of cases) (Table 3, Figure 1).

Other placental findings in this study were marginal insertion of the umbilical cord (in 6% of cases), hypercoiling of the umbilical cord (10% of cases) and phagocytosis of the meconium (in 16% of cases).

## 4. Discussion

Although there have been many reports of babies born to mothers testing positive for COVID-19, in our study, no case of vertical transmission was seen. As other studies have shown, there are only rare case reports with probable vertical transmission [6,13,14,15,18,31]. This fact suggests either that SARS-CoV-2 rarely infects the placenta, or that the placenta functions as a barrier despite its infection. In our study, all but one patient was diagnosed with COVID-19 in the peripartum period by RT-PCR. Even though the number of COVID-19 cases was high in our country, it was noted that most patients (84%) were asymptomatic. Because most patients were asymptomatic at the time of the diagnosis, we could not know how many days the infection had been present for, so it is possible that the placental histopathological findings had been present for a longer period.

Judging by the fact that in our clinic there are many patients with only a few or no medical visits during pregnancy and that during the pandemic the number of these cases was even higher, it is safe to say that the follow-up care was insufficient. In our group, 15 of the women had no antenatal care, whereas the mean number of follow-up examinations per pregnancy was 4.4. With a mean birth weight of 3046.8 g and a mean Apgar score of 9 at 1 min, 24% of newborns were admitted to NCIU in the first 24 h after birth. Admission to NCIU was needed because of acute neonatal respiratory distress and neonatal sepsis, with one case of aspiration pneumonia. We do not know whether these complications were in correlation with SARS-CoV-2 infection, but it is safe to say that the evolution of these newborns was caused by the lack of antenatal care.

As other studies have shown, the microscopic pathological aspects are divided into four subcategories: maternal-side signs of vascular malperfusion, foetal-side signs of vascular malperfusion, inflammatory changes and other placental findings [2,3,4,21,27,28]. Pathological changes in the placentas were acute or subacute, since, in this study, we included only patients with acute COVID-19. In our study, most frequent maternal-side signs of vascular malperfusion are increased perivillous fibrin deposition, increased microcalcifications and intervillous thrombosis, as was seen in other studies [19,20,32,33]. The results were similar to those found in studies published by Giordano et al. [2] and Shanes et al. [19], where the intervillous thrombi were the most common. These lesions are frequent in cases of COVID-19 and may be related to hypercoagulability in the intervillous space [2,19,34]. The most common sign of foetal vascular malperfusion is the presence of avascular villi, which are considered the consequence of an obstruction of large vessels in the placenta and are due to hypercoagulation, cardiac dysfunction, hypoxia, long hyper-coiled cords and abnormal umbilical cord marginal insertion [2,22,35]; in this study, similar findings were identified. The most frequent inflammatory change is villitis, although it was not as commonly identified in the placentas that came from SARS-CoV-2-positive pregnant women. These results were similar to the ones found in the study by Hecht et al. [8]. As other studies have shown, the microscopic pathological signs identified at the level of the placenta are not specific for SARS-CoV-2 infection, but for almost any hypoxic pathology and viral infection [2,19,20,21,25,26,27,28,29,30].

Our morphological study was limited in that we did not use optimal controls. The best controls would have been placentas delivered by women with COVID-19 symptoms (e.g., hypoxia) but a negative SARS-CoV-2 test. This should be done to definitively identify if coagulopathic or inflammatory pathologies are increased in this population. The fact that 84% of patients were asymptomatic was another important limitation of our study; a recent systematic review showed that the severity of the infection plays a key role in pregnancy outcome [9].

## 5. Conclusions

In conclusion, the histopathological aspects found in placentas that came from COVID-19-positive pregnant women are common for many other diseases, but when they are found together, they are highly suggestive of viral infectious involvement of the placenta associated with hypoxia. Microvasculopathy, which manifests mostly as signs of maternal malperfusion, is a common finding in placentas of SARS-CoV-2-positive patients.

## Figures and Tables

**Table 1:** Maternal characteristics.

Variables	50 Cases (100%)	
Age (years)	26.88 (15–43) Median–26	
Gravidity	3.06 (1–9) Median–2.5	
Parity	2.02 (1–7) Median–2	
No. of (follow up) examinations per pregnancy	4.4 (0–12) Median–4	
Gestational age at the moment of RT-PCR SARS-CoV-2 positive result (weeks)	38.12 (27–41) Median–38	
Interval between RT-PCR SARS-CoV-2 positive result (COVID-19 diagnosis) and birth (days)	0.94 (0–10) Median–0	
COVID-19 severity of illness category	Asymptomatic	42 (84%)
	Mild	7 (14%)
	Moderate	0
	Severe	1 (2%)
Gestational age at delivery (weeks)	38.18 (27–41) Median–38	
Type of delivery	Spontaneous	30 (60%)
	Caesarean section	20 (40%)
Pregnancy-related complications (cases)	Gestational hypertension	3 (6%)
	Foetal growth restriction	1 (2%)
	Gestational diabetes	1 (2%)
	Rh incompatibility with isoimmunisation	2 (4%)
	Genital infection	2 (4%)
	No complications	41 (82%)
Level of Haemoglobin (g/dL)	11.85 (8.4–14.50) Median–11.70	
Leucocytes (×10^9^/L)	10.90 (6.23–15.66) Median–11.28	
Thrombocytes (×10^9^/L)	255.54 (114–426) Median–261	
C-reactive protein (mg/L)	53.79 (2.34–198.30) Median–29.40	
Coagulation abnormalities (cases)	Present	3 (6%)
	Absent	47 (94%)
Blood group	0 (I)	16 (32%)
	A (II)	12 (24%)
	B (III)	15 (30%)
	AB (IV)	7 (14%)
Rh	Positive	45 (90%)
	Negative	5 (10%)

**Table 2:** Newborns’ characteristics.

Variables	50 Cases (100%)	
Birth weight (grams)	3046.8 (690–3800) Median–3075	
Size for Gestational Age	SGA	2 (4%)
	AGA	48 (96%)
	LGA	0
Apgar score 1 min	9 (3–10) Median–9	
Amniotic fluid	Clear	42 (84%)
	Meconium stained	8 (16%)
Admission to NCIU in the first 24 h after birth	Yes	12 (24%)
	No	38 (76%)
Newborn days of life at discharge	6.86 (3–17) Median–5	
Clinical course	No complications	23 (46%)
	Intense newborn jaundice	2 (4%)
	Bacterial sepsis	3 (6%)
	Respiratory distress syndrome	8 (16%)
	Aspiration pneumonia	1 (2%)
	Neonatal death	1 (2%)
	Lost to follow up	12 (24%)

**Table 3:** Placental characteristics.

Diagnosis	Placentas from COVID-19-Positive Women 50 Cases (100%)	
Placental weight (grams)	509 (115–633) 512	
Macroscopic aspect	Normal	41 (82%)
	Meconium stained	8 (16%)
	Calcar deposits	1 (2%)
Maternal vascular malperfusion (MVM)		
Infarction	15 (30%)	
Increased perivillous fibrin deposition	32 (64%)	
Accelerated villous maturation	18 (36%)	
Tenney-Parker change	19 (38%)	
Decidual arteriopathy	10 (20%)	
Intervillous thrombosis	23 (46%)	
Increased microcalcifications	28 (56%)	
Foetal vascular malperfusion (FVM)		
Thrombi in the foetal circulation	16 (32%)	
Avascular villi	36 (72%)	
Karyorrhexis	11 (22%)	
Delayed villous maturation	17 (34%)	
Chorangiosis	10 (20%)	
Inflammatory changes		
Villous oedema	12 (24%)	
Chorioamniotitis	7 (14%)	
Subchorionitis	9 (18%)	
Chronic Villitis	16 (32%)	
Chronic deciduitis	7 (14%)	
Other placental findings		
Marginal insertion of the umbilical cord	3 (6%)	
Hypercoiling of the umbilical cord	5 (10%)	
Phagocytosis of meconium	8 (16%)	

## Data Availability

The data presented in this study are available on request from the corresponding author.
